# Achilles tendon and triceps surae muscle properties in athletes

**DOI:** 10.1007/s00421-023-05348-4

**Published:** 2023-11-11

**Authors:** Maria Sukanen, Ra’ad M. Khair, Johanna K. Ihalainen, Iida Laatikainen-Raussi, Pauline Eon, Antoine Nordez, Taija Finni

**Affiliations:** 1 Faculty of Sport and Health Sciences, University of Jyväskylä, Jyväskylä, Finland; 2 Nantes Université, Movement-Interactions-Performance, MIP, UR 4334, F-44000 Nantes, France; 3 Institut Universitaire de France, Paris, France

**Keywords:** Mechanical properties, Tendon non-uniformity, Ultrasound speckle tracking, Shear wave elastography, Stiffness

## Abstract

**Purpose:**

The aim of this study was to investigate internal Achilles tendon (AT) displacement, AT shear wave velocity (SWV), and triceps surae (TS) muscle shear modulus in athletes.

**Methods:**

Internal AT displacement was assessed using ultrasound during isometric contraction. Shear wave elastography was used to assess AT SWV (m × s^–1^) at rest and TS muscle shear modulus (kPa) during passive ankle dorsiflexion.

**Results:**

A total of 131 athletes participated in this study. Athletes who had not exercised within two days had greater AT non-uniformity and mean anterior tendon displacement, and lower SWV at the proximal AT measurement site (mean difference [95% CI]: 1.8 mm [0.6–2.9], *p* = 0.003; 1.6 mm [0.2–2.9], *p* = 0.021; – 0.9 m × s^–1^ [– 1.6 to – 0.2], *p* = 0.014, respectively). Male basketball players had a lower mean AT displacement compared to gymnasts (– 3.7 mm [– 6.9 to – 0.5], *p* = 0.042), with the difference localised in the anterior half of the tendon (– 5.1 mm [– 9.0 to – 1.1], *p* = 0.022). Male gymnasts had a smaller absolute difference in medial gastrocnemius-minus-soleus shear modulus than basketball players (59.6 kPa [29.0–90.2], *p* < 0.001) and track and field athletes (52.7 kPa [19.2–86.3], *p* = 0.004). Intraclass correlation coefficients of measurements ranged from 0.720 to 0.937 for internal AT displacement, from 0.696 to 0.936 for AT SWE, and from 0.570 to 0.890 for TS muscles.

**Conclusion:**

This study provides a reliability assessment of muscle and tendon SWV. The relative differences in passive TS muscle shear modulus suggest sport-specific adaptation. Importantly, in healthy individuals, lower AT displacement after exercise may reflect the time required for tendon recovery.

**Supplementary Information:**

The online version contains supplementary material available at 10.1007/s00421-023-05348-4.

## Introduction

The mechanical properties of musculoskeletal structures are critical for athletic performance (Bojsen-Møller et al. 2005), and understanding muscle–tendon mechanics is important in clinical settings and in research on sports-related conditions (Arampatzis et al. 2020). During locomotion, the Achilles tendon (AT) can be subjected to considerable mechanical loads while transmitting forces from the triceps surae (TS) muscle (Komi et al. 1992; Finni et al. 2000). Tendons adapt to long-term mechanical loading through increased physical exercise (Bohm et al. 2015), resulting in optimised force transmission and injury resistant tissue (Kjær 2004). For example, sprinters are shown to have higher AT stiffness (Arampatzis et al. 2007) and endurance runners larger AT cross-sectional area compared to controls (Magnusson and Kjaer 2003). Despite the adaptive changes, the AT and TS muscles are common sites of musculoskeletal conditions, with the vast majority originating from sports activities (Lagas et al. 2020; Pedret et al. 2020; Leino et al. 2022). Although these structures have been extensively studied, knowledge about their biomechanical properties and adaptation remains incomplete.

Achilles tendon is formed by three subtendons originating from the medial gastrocnemius (MG), lateral gastrocnemius (LG), and soleus (SOL) muscles. The subtendons twist towards their insertion at the calcaneal tuberosity (Edama et al. 2015; Pękala et al. 2017), forming the complex structure of the AT (Ekiert et al. 2021). Differential forces (Arndt et al. 1999) are exerted on the subtendons, resulting in non-uniform sliding within the AT (Arndt et al. 2012; Bojsen-Møller and Magnusson 2019). This non-uniformity is considered a sign of healthy tendon function and can be investigated using the speckle tracking method, where ultrasound recordings of the tendon can be tracked during force production or passive movement to detect internal tendon displacement (Slane and Thelen 2014). Studies have shown that the anterior regions of the tendon have greater displacement than the posterior regions (Slane and Thelen 2014; Stenroth et al. 2019; Khair et al. 2021), and the overall non-uniformity appears to decrease with age (Slane and Thelen 2015; Franz and Thelen 2016) and after injury (Beyer et al. 2018; Khair et al. 2021). To date, studies have comprised general population, and athlete adaptations in AT internal displacement have not been investigated. As the internal AT displacement has successfully distinguished healthy and injured individuals (Beyer et al. 2018; Khair et al. 2021), it may serve as an indicator of AT health in the athletic population.

Stiffness is a mechanical property of muscles and tendons that represents the resistance of the tissue to deformation and is an important determinant of force production and performance (Roberts 2016). Stiffness is commonly determined from the force–elongation relationship by measuring the force and elongation of the tracked structure. The three TS muscles have differential passive stiffness, which may be altered as the contractile and connective tissues within the muscle adapt (Fouré et al. 2011; Longo et al. 2021). Regarding the tendon, high AT stiffness may enable fast force development (Waugh et al. 2013; Hirayama et al. 2017), while optimally compliant AT allows maximal movement efficiency (Lichtwark and Wilson 2008). Therefore, assessing passive AT and TS muscle stiffness in different athletes may improve our understanding on sport-specific requirements and adaptations.

Shear wave elastography (SWE) is an emerging ultrasound technique for assessing the viscoelastic properties of soft tissues. This method provides a measurement of shear wave velocity (m/s), which can be further expressed as shear modulus and serve as a surrogate quantification of passive tissue stiffness (Bercoff et al. 2004). SWE has been validated ex vivo against conventional tensile testing of skeletal muscle (Eby et al. 2013) and the tangent traction modulus of tendon calculated using a material testing system (Zhang and Fu 2013). Several publications have reported its reliability in assessing muscle (e.g. Lacourpaille et al. 2012) and tendon stiffness (Schneebeli et al. 2021). SWE provides a direct estimate of muscle force (Bernabei et al. 2020) and the increase in muscle tension due to stretching (Maïsetti et al. 2012; Le Sant et al. 2017). In tendons, SWE is associated with in vivo dynamometry and ultrasound-based tendon stiffness only over small strains up to 10% of maximal voluntary contraction, suggesting that SWE is an appropriate assessment method in resting rather than loading conditions (Mifsud et al. 2023). When assessing muscle and tendon properties using SWE, previous studies have explored outcomes between individuals regarding sex (Chino and Takahashi 2016; Fouré et al. 2022), injury history (Saeki et al. 2018; Dirrichs et al. 2018;), and the effect of both short-term exercise (Payne et al. 2018a) and long-term training (Avrillon et al. 2020; Miyamoto et al. 2019). To determine the correct interpretation of the SWE technique, it is still necessary to expand the understanding of normal tissue-specific variation between individuals from different athletic groups and according to their physical status.

Therefore, the aim of this study was to acquire a large dataset of internal AT displacement and passive AT shear wave velocity and TS muscle shear modulus in athletes from different sports. Furthermore, we investigated whether (1) previous exercise, (2) history of lower limb injury, and (3) individual characteristics of sex and sport specialisation are factors that distinguished AT displacement or passive AT shear wave velocity and TS muscle shear modulus. We expected to find differences between the groups due to previous exercise, injury history, and long-term sport-specific loading. In addition, we used linear regression to examine whether there are significant predictors of internal AT displacement among the measured variables with the hypothesis that AT shear wave velocity and sport specialisation would influence the internal AT displacement. This study also investigated the reliability of SWE and internal AT displacement measurements.

## Materials and methods

### Athlete recruitment

Young competitive athletes were recruited from local sports academies between 2018 and 2021. Inclusion criterion was active participation in competitive sport at a national level (Tier 3 or higher, McKay et al. 2022). Exclusion criteria were previous injuries or health conditions that prevented active participation in sport within 6 months. A total of 131 young athletes were eligible for this study. Participants were asked about participation in exercise during the previous two days, previous lower limb injuries, age, sex, sports specialisation, and leg dominance. Leg dominance was determined by the preferred leg used to kick a ball. Height and weight were measured, and body mass index (BMI) was calculated. Ethical approval was obtained from the Research Ethics Committee of the Central Finland Health Care District (5U/2019), and all study procedures were conducted according to the Declaration of Helsinki. Each participant provided a signed informed consent prior to participation. For underaged study participants, signed informed consent was also obtained from their legal guardian.

Athletes were grouped according to previous exercise participation and lower limb injury. For previous exercise, two subgroups were formed depending on whether the athletes had exercised within two days. For previous injury, two subgroups were formed depending on whether the athletes had sustained a lower limb injury within 6 months.

### Shear wave elastography measurement

Supersonic shear wave elastography (Aixplorer Supersonic Imagine, v. 12.3.1 Aix-en-Provence, France) was used to estimate passive AT and TS muscle stiffness. The technique has been described in detail previously (Bercoff et al. 2004) and it yields tissue shear wave velocity (SWV) and shear modulus (μ), of which the latter is calculated using the formula: *μ* = *ρV*^2^ (*ρ* = muscle mass density 1000 kg m^3^) (Bercoff et al. 2004; Gennisson et al. 2003). A 38 mm linear transducer (2–10 MHz, SL10-2) was used to record AT SWV and SOL shear modulus, and a 50 mm linear transducer (5–18 MHz, SL18-5) to image MG and LG shear modulus. All measurements were performed with a custom musculoskeletal pre-set (penetration mode, smoothing level 5, persistence off, opacity 100%) with the probe parallel to the muscle and tendon fascicles and perpendicular to the skin. Probe alignment was determined when multiple muscle or tendon fascicles and both superficial and deep aponeurosis were visible in the B-mode image. Pressure between the probe and skin was kept to a minimum. All recordings were taken from both legs in random order.

Intra- and interrater reliability of laboratory measurements were tested in accordance with the study protocol for two raters (*n* = 8). Intrarater measurements were carried out with approximately 15 min between two trials. Raters were not blinded to each other’s recordings for interrater assessment. All reliability measurements were performed during a single laboratory visit by pilot participants matched to the study sample.

### Data collection

**Fig. 1:**
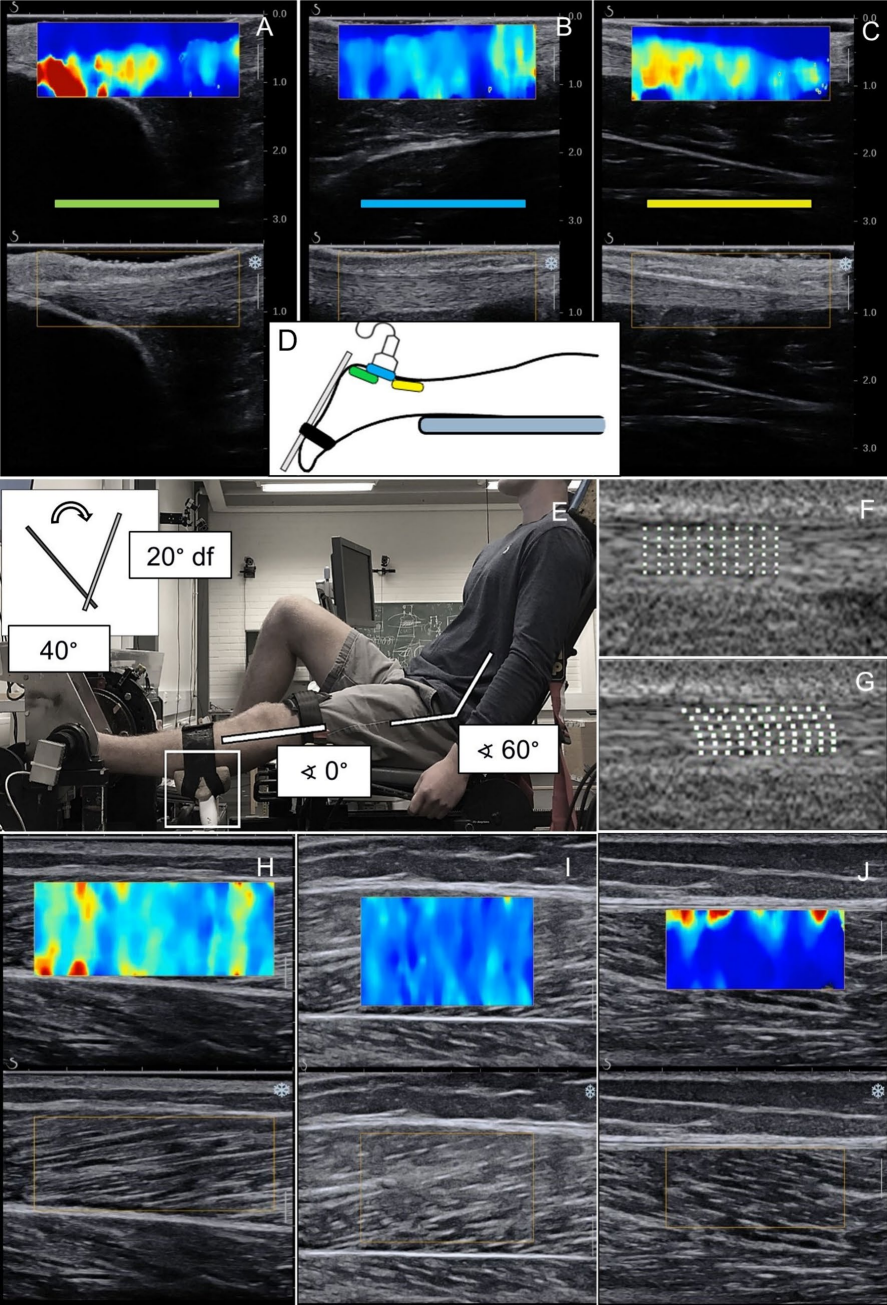
Measurement protocol for AT shear wave velocity, TS muscle shear modulus, and AT displacement**. a–c** SWE and B-mode images of AT at three different measurement sites; **d** representation of the SWE imaging sites of AT; **e** participant position on the ankle dynamometer when **f–g** internal AT displacement was assessed during submaximal isometric contraction, and when **h–j** SWE of the medial gastrocnemius, lateral gastrocnemius, and soleus muscle was measured during passive ankle dorsiflexion. Example in **j** shows that although the SWE region of interest was initially placed in the middle of the muscle, the changes in muscle position during dorsiflexion caused the region to overlap the aponeurosis, which shows higher shear modulus in the upper part of the image. In such a case, the region of interest for analysis was chosen not to include the affected values

For the resting AT SWE examination, participants were lying prone with both feet fixed at 25° ankle plantarflexion. The SWE elasticity range was set from 0 to 800 kPa and the image depth was set to ~ 2.5 cm. Using B-mode ultrasound, the proximal head of calcaneus was identified and marked on the skin. A second mark was placed three centimetres proximally. The SWE map of the AT was recorded longitudinally from three distal locations: first, with the proximal head of the calcaneus aligned in the centre of the image (ATdist), and then moving the transducer 18 mm (ATmid) and 48 mm (ATprox) proximally from the first location. The region of interest was set as wide as possible to cover the entire AT. The transducer was held still for ~ 5 s during each recording (Fig. 1a–d).

Triceps surae SWE imaging locations were determined using B-mode ultrasound and marked for longitudinal probe placement with the participants lying prone with both feet relaxed over the end of measurement table. For the gastrocnemius, the area of greatest muscle bulk was used. Soleus was imaged laterally from the area where the muscle lies superficially, distal to LG muscle–tendon junction. Next, participants were seated on a custom-built ankle dynamometer with the hip in 60° flexion and the knee fully extended (Fig. 1e). The foot was firmly attached to the dynamometer footplate and the knee was secured in extension with an inelastic strap. The axis of the dynamometer was aligned with the presumed centre of rotation of the ankle. The neutral ankle position (0°) was considered as the foot was perpendicular to the leg. Torque and ankle angle data were sampled at a frequency of 1 kHz using a 16-bit analogue-to-digital converter (Power 1401, Cambridge Electronic Design, Cambridge, UK) connected to a computer. Torque and angle data were recorded using Spike2 software (v. 6.17, Cambridge Electronic Design Limited, Cambridge, UK). Participants were instructed to remain as relaxed as possible, when the ankle joint was rotated at 2°/s from 40° plantarflexion to a maximum of 20° dorsiflexion. Each participant was familiarised with the movement prior to recording. If participants reported discomfort during the stretch towards dorsiflexion or if the simultaneously observed torque level exceeded a set limit of 15 Nm, a lower range of motion of 15° dorsiflexion was used. This range could be further reduced to 10° if necessary. Elasticity range was set to a maximum of 0 to 600 kPa and image depth was adjusted according to the individual muscle thickness. For conditioning, participants performed repetitions of submaximal isometric contractions. After ensuring appropriate B-mode visibility, the US transducer was fixed to the calf using a custom probe holder and an elastic strap, and the passive movements were repeated while imaging MG, LG, and SOL in random order (Fig. 1h–j). To synchronise the data, a continuous transistor–transistor logic pulse was sent from the ultrasound device to the analogue–digital converter to mark the duration of each SWE recording. Participants were given one minute rest between measurements. The absence of muscle activation was monitored visually by ultrasound and real-time torque data.

Finally, the internal displacement of the AT was recorded during submaximal voluntary contraction. Participants were seated on the ankle dynamometer as described previously, with ankle joint at 0° and the US transducer placed longitudinally on the distal AT. Participants were first familiarised with isometric plantarflexion ramp contractions up to a target torque of 50 Nm. After familiarisation, a video of B-mode images during the contraction was recorded and stored for offline analysis (Fig. 1f, g).

### Data processing

Shear wave elastography recordings were processed using a custom software developed for MATLAB (ElastoGUI, v. R2021b, MathWorks Inc, Natick, MA, USA) with semi-automatic processing. Each pixel of the SWE colour maps was converted to SWV and then to shear modulus based on the recorded colour scale. For each SWE recording, the area of analysis was adjusted to cover the largest possible area within the superficial and deep aponeurosis while avoiding artefacts. For AT, acceptable saturation and void levels of < 3% and < 0.1% were used, and for TS muscles the limits were set to < 1%. In ATdist, SWV variation caused by the calcaneus was excluded (see Fig. 1a). In TS muscle SWE processing, SWV variation due to reflection from the aponeurosis was excluded to ensure that the assessment was restricted to muscle tissue only (e.g. Fig. 1j). In results, AT SWE measurements are reported as mean SWV (m × s^–1^). TS muscle SWE measurements are given as mean peak shear modulus (kPa) to represent the elastic properties of the muscle under passive stretch loading. All SWE data are provided in kPa and as SWV values in the Supplementary Information (SI1) to facilitate global scientific data comparison. Absolute differences in passive shear modulus between TS muscles (MG-LG, MG-SOL, LG-SOL) were also calculated.

Mean (SD) values of the analysed areas of the three muscles and the three AT SWE measurement locations were as follows: SOL 0.8 cm^2^ (0.5); MG 2.1 cm^2^ (0.7); LG 2.0 cm^2^ (0.7); ATdist 0.3 cm^2^ (0.1); ATmid 0.9 cm^2^ (0.2); ATprox 0.7 cm^2^ (0.2), respectively. Saturation was present in 124 analysed AT SWE recordings (average saturation % in different locations: ATdist 0.7%; ATmid 0.7%; ATprox 0.6%).

Changes in TS muscle shear modulus during passive ankle range of motion were determined to differentiate the muscle-specific relationship during passive stretch. In addition, we calculated the percentage contribution of each TS muscle from the total summed TS muscle shear modulus to describe the contribution of each muscle to resistance during the passive range of motion. Torque signal was filtered using a fourth order Butterworth low-pass filter with a cut-off frequency of 8 Hz.

Internal AT displacement was assessed in MATLAB (v. R2021b, MathWorks Inc, Natick, MA, USA) using 2D speckle tracking (Slane and Thelen 2014). The region of interest was manually positioned over the distal tendon with its size individually adjusted to ensure that only tendinous tissue was included. Within the region of interest, a grid of six locations across the thickness of the tendon and 11 locations across the length of the tendon was generated (e.g. Fig. 1f, g). Each tracking was visually monitored to ensure the quality of data processing. The mean displacement (mm) was calculated as the average displacement across the six tracked tendon locations. Mean displacement was further examined by dividing the tendon into anterior and posterior halves (Franz et al. 2015). This was done to assess whether any adaptations were localised to different parts of the AT. Non-uniformity was calculated as maximum-minus-minimum displacement within the tracked locations across the tendon.

### Statistical analysis

All statistical analyses were performed with IBM SPSS Statistics for Windows (v.28.0, Armonk, NY) and visualised using JASP (JASP v. 0.16.4, Amsterdam, The Netherlands) and Microsoft Excel (Excel for Microsoft 365 v. 2209). All descriptive statistics are presented as means and standard deviations (SD). Statistical significance was defined as *P* < 0.05. The normality of variables was assessed using the Shapiro–Wilk *W* test. The 95% confidence intervals (CI) for statistical tests are reported when appropriate.

We first tested whether the outcome variables differed between legs according to leg dominance. Since paired samples *t* test showed no significant differences, we chose to use only dominant leg in the analysis and results.

Independent samples *t* test was used to determine mean differences in AT displacement outcomes and passive AT SWV and TS muscle shear modulus according to previous exercise participation, injury history, and sex. Paired samples *t* test was calculated to detect differences in SWV between AT SWE measurement locations, and the shear modulus between each TS muscle. Analysis of variance (ANOVA) was used to evaluate differences in AT displacement outcomes and passive AT SWV and TS muscle shear modulus between sports specialisations. Bivariate correlations were calculated between AT displacement outcomes and passive AT SWV and TS muscle shear modulus. Non-parametric tests were used for ATdist variable due to non-normal distribution. Multiple linear regression analysis was performed to assess whether age, sex, sport, or passive AT SWV or TS muscle shear modulus predicted maximal AT non-uniformity. A natural logarithm transformation was calculated for the model for ATdist.

The intra- and interrater reliability of laboratory measurements were determined using the intraclass correlation coefficient (ICC_3,1_), the coefficient of variation (CV), the standard error of measurement (SEM), and the minimum detectable change (MDC).

## Results

The general characteristics of the athletes are shown in Tables 1, 2 presents descriptive characteristics of the AT internal displacement outcomes, and AT shear wave velocity and TS muscle shear modulus.

**Table 1:** Characteristics of the study population

Demographic	*N* = 131	Males (*n* = 48)	Females (*n* = 83)
Age, mean (SD)	19.5 (3.8)	18.8 (3.1)	19.9 (4.1)
BMI, mean (SD)	23.1 (2.7)	22.7 (2.7)	23.3 (2.8)
Height, mean (SD)	173.6 (11.6)	184.9 (9.8)	167.2 (6.4)
Weight, mean (SD)	69.9 (13.3)	79.2 (14.8)	64.7 (8.9)
Sport, *n* (%)			
Football	31 (23.7)	–	31 (37.4)
Ice hockey	27 (20.6)	–	27 (32.5)
Basketball	27 (20.6)	27 (56.2)	–
Track and field	21 (16.0)	14 (29.2)	7 (8.4)
Gymnastics	25 (19.1)	7 (14.6)	18 (21.7)
Leg dominance, *n* (%)			
Right	85 (64.9)	21 (43.8)	64 (77.1)
Left	46 (35.1)	27 (56.2)	19 (22.9)
Injuries within 6 months, *n* (%)			
Injured	36 (27.5)	15 (31.2)	21 (25.3)
Not injured	95 (72.5)	33 (68.8)	62 (74.7)
Previous exercise, *n* (%)			
Exercise within 2 days	80 (61.1)	20 (41.7)	23 (27.7)
No exercise	51 (38.9)	28 (58.3)	60 (72.3)

*n* number of participants, *SD* standard deviation, *BMI* body mass index

**Table 2:** Descriptive values of Achilles tendon non-uniformity and mean displacement (mm), triceps surae muscle shear modulus (kPa), and Achilles tendon shear wave velocity (m × s^–1^) of the dominant leg

Subgroup	AT internal movement (mm)		Shear modulus (kPa)			Shear wave velocity (m × s^–1^)		
	AT non-uniformity (SD)	AT mean displacement (SD)	MG peak mean (SD)	LG peak mean (SD)	SOL peak mean (SD)	ATdist mean (SD)	ATmid mean (SD)	ATprox mean (SD
All athletes	7.6 (3.3)	8.9 (3.1)	90.7 (23.3)	53.4 (16.4)	22.9 (13.4)	7.5 (1.8)	9.3 (2.3)	10.7 (2.0)
Males	7.8 (3.6)	8.8 (3.2)	87.3 (22.4)	53.4 (16.4)	21.5 (13.7)	7.6 (2.0)	9.6 (2.6)	10.6 (2.3)
Females	7.5 (3.1)	8.9 (3.0)	92.7 (23.8)	53.5 (16.5)	23.5 (13.3)	7.3 (1.6)	9.1 (2.1)	10.8 (1.8)
Sport								
Football	7.4 (2.6)	8.9 (2.7)	93.8 (22.5)	55.3 (14.3)	27.5 (17.3)	7.8 (2.0)	8.6 (1.9)	10.6 (1.7)
Ice hockey	7.5 (3.2)	8.5 (3.1)	90.3 (28.8)	54.5 (17.9)	21.0 (10.9)	6.8 (1.3)	9.1 (2.1)	10.8 (1.8)
Basketball	6.6 (2.4)	7.9 (2.4)	90.3 (21.9)	56.7 (16.1)	18.4 (9.1)	7.6 (2.0)	9.3 (2.6)	10.5 (2.5)
Track and field	7.6 (4.3)	8.4 (3.2)	89.2 (19.7)	47.1 (15.7)	20.9 (10.5)	7.8 (1.9)	10.0 (2.4)	11.4 (1.9)
Gymnastics	8.8 (3.5)	10.4 (3.5)	89.0 (23.7)	52.3 (17.8)	25.0 (15.2)	7.3 (1.7)	9.5 (2.3)	10.5 (2.2)

*AT* Achilles tendon, *MG* medial gastrocnemius, *LG* lateral gastrocnemius, *SOL* soleus, *ATdist*, *ATmid*, *ATprox* distal, middle, and proximal AT SWE imaging sites, *SD* standard deviation

Reliability of internal AT displacement measurements (Table 3) ranged from ICC 0.720 to 0.907 for intrarater, and from 0.741 to 0.937 for interrater assessment. MDC showed a large variation of up to 2.2 mm and 4.1 mm for AT non-uniformity and mean displacement. Reliability of SWE measurements showed intrarater ICC’s between 0.570 and 0.936 and interrater ICC’s between 0.687 and 0.866 with large variation in 95% confidence intervals. CV and MDC of SWE measurements were ~ 10% and ~ 1.5 m × s^−1^ for AT, ~ 10% and ~ 20 kPa for MG, ~ 12% and ~ 13 kPa for LG, and up to 40% and ~ 15 kPa for SOL.

**Table 3:** Intra- (A) and interrater (B) reliability of laboratory measurements (n = 8)

Location	Rater	Mean (SD) (mm/ m × s^–1^/kPa)	ICC (90% CI)	SEM (mm/m × s^–1^/kPa)	MDC (mm /m × s^–1^/kPa)	CV%
A						
AT non-uniformity (mm)	1	5.1 (2.1)	0.859 (0.392–0.974)	0,8	2,2	18.7
	2	4.1 (1.0)	0.907 (0.561–0.983)	0.3	0.9	6.5
AT mean displacement (mm)	1	6.1 (2.8)	0.720 (0.032–0.945)	1.5	4.1	41.6
	2	6.6 (2.4)	0.777 (0.294–0.944)	1.1	3.1	13.3
ATdist (m × s^–1^)	1	7.8 (1.4)	0.715 (0.020–0.944)	0.9	2.6	10.1
	2	8.0 (1.3)	0.884 (0.475–0.979)	0.5	1.3	6.0
ATmid (m × s^–1^)	1	10.0 (1.8)	0.909 (0.570–0.984)	0.6	1.5	5.4
	2	10.4 (2.1)	0.936 (0.679–0.989)	0.5	1.5	6.0
ATprox (m × s^–1^)	1	10.3 (1.8)	0.909 (0.567–0.984)	0.5	1.5	5.6
	2	10.8 (1.2)	0.801 (0.221–0.962)	0.5	1.5	5.2
MG (kPa)	1	76.1 (17.5)	0.890 (0.417–0.984)	6.0	16.7	7.6
	2	87.2 (20.5)	0.811 (– 0.250 to 0.987)	9.4	26.2	13.3
LG (kPa)	1	40.8 (8.2)	0.686 (– 0.138 to 0.949)	4.6	12.8	11.7
	2	41.0 (7.8)	0.696 (– 0.119 to 0.951)	4.4	12.2	11.9
SOL (kPa)	1	25.1 (9.7)	0.570 (– 0.321 to 0.926)	6.5	17.9	40.5
	2	25.3 (7.5)	0.607 (– 0.401 to 0.950)	4.9	13.5	26.0
B						
AT non-uniformity (mm)		4.5 (0.9)	0.741 (– 0.175 to 0.969)	0.4	1.2	4
AT mean displacement (mm)		5.8 (1.9)	0.937 (0.526–0.993)	0.5	1.3	8.0
ATdist (m × s^–1^)		7.9 (1.4)	0.713 (0.336–0.893)	0.7	2.0	9.4
ATmid (m × s^–1^)		10.2 (1.9)	0.837 (0.583–0.942)	0.8	2.2	7.7
ATprox (m × s^–1^)		10.6 (1.5)	0.696 (0.305–0.886)	0.8	2.3	8.1
MG (kPa)		77.2 (18.6)	0.866 (0.636–0.955)	6.7	18.4	9.7
LG (kPa)		40.5 (7.9)	0.777 (0.439–0.922)	3.7	10.4	9.1
SOL (kPa)		25.1 (8.7)	0.687 (0.270–0.887)	4.9	13.7	26.1

*n* number of participants, *SD* standard deviation, *ICC* intraclass correlation coefficient, *SEM* standard error of measurement, *MDC* minimal detectable change, *CV* coefficient of variation, *AT* Achilles tendon, *ATdist*, *ATmid*, *ATprox* distal, middle, and proximal AT SWE imaging sites, *MG* medial gastrocnemius, *LG* lateral gastrocnemius, *SOL* soleus

### Differences between groups

**Fig. 2:**
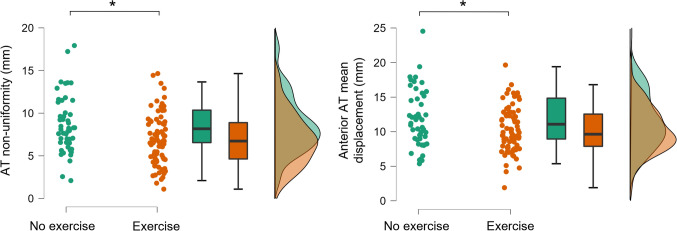
Changes in Achilles tendon non-uniformity (mm) and anterior tendon mean displacement (mm) between participants who had or had not exercised within two days prior to measurement. Significance of group differences is marked with an asterisk*

When examining groups with or without exercise, exercise within two days prior to assessment resulted in significant differences for internal AT displacement and ATprox SWV between groups. AT non-uniformity and mean anterior tendon displacement were greater in athletes who had not exercised before the measurements (mean difference [95% CI]: 1.8 mm [0.6–2.9], *p* = 0.003; 1.6 mm [0.2–2.9], *p* = 0.021, respectively) (Fig. 2). Also, ATprox SWV was lower in athletes without previous exercise (– 0.9 m × s^–1^ [– 1.6 to – 0.2], p = 0.014).

No statistically significant differences between groups were found in any of the biomechanical outcomes based on history of lower limb injury, sport specialisation, sex, or leg dominance.

### Regional differences in SWE outcomes

**Fig. 3:**
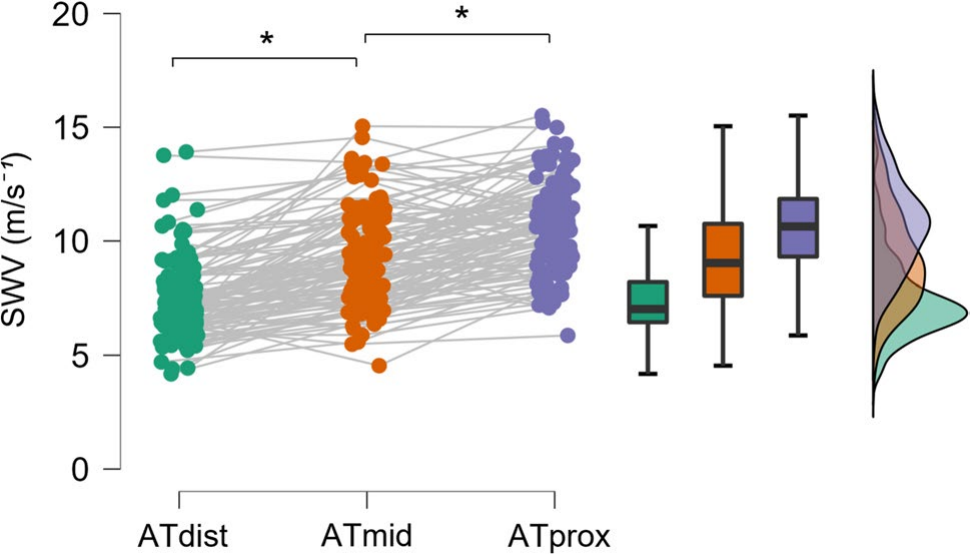
Regional differences in AT shear wave velocity across the distal tendon. *SWV* shear wave velocity (m × s^–1^); *p < 0.001, *ATdist* distal AT location, *ATmid* middle AT location, *ATprox* proximal AT location. Significance of mean differences is marked with an asterisk*

**Fig. 4:**
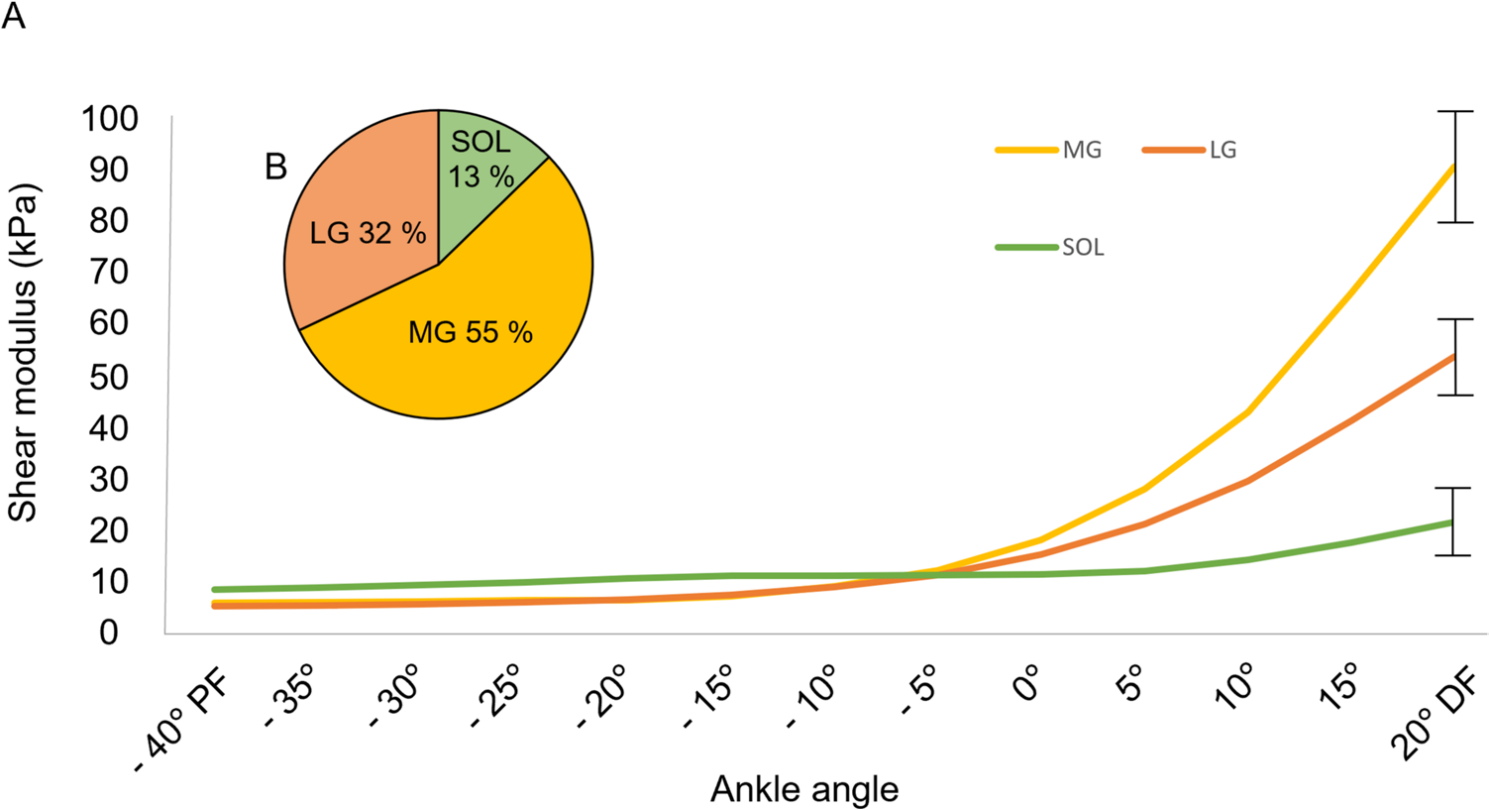
**a** The change in passive shear modulus of SOL, MG, and LG during passive ankle range of motion; and **b** the percentage of each muscle head to the total summed TS shear modulus at 20° of passive ankle dorsiflexion. Data are averaged across the entire sample

AT SWV showed significant regional differences between each measurement location (*p* < 0.001) with increasing velocities from distal to proximal orientation (Fig. 3). Shear modulus of the TS muscle showed significant (*p* < 0.001) differences between SOL, MG, and LG during the passive range of motion (Fig. 4a). The change in shear modulus was greatest in MG, followed by LG and SOL. The contribution (%) of SOL, MG, and LG to total TS muscle shear modulus during the passive ankle range of motion to 20° dorsiflexion is shown in Fig. 4b.

### Correlations

Bivariate correlations showed only a negligible association between LG shear modulus and AT mean displacement (*r* = – 0.185, *p* = 0.041), absolute MG-LG shear modulus difference and AT mean displacement (*r* = 0.195, *p* = 0.033), and between MG shear modulus and ATprox SWV (*r* = 0.223, *p* = 0.013).

### Regression analysis

A multiple linear regression model was computed to test whether age, sex, sport, TS muscle shear modulus, or AT SWV associate with maximal AT non-uniformity. The model was not significant (*R*^2^ = 0.133, *F* = 0.815, *p* = 0.605), and none of the predictor variables predicted maximal AT non-uniformity.

## Discussion

We explored the properties of the Achilles tendon and triceps surae muscle in young competitive athletes from different sports, presenting values assessed with ultrasound speckle tracking and shear wave elastography. Exercise within two days before measurement was found to affect Achilles tendon non-uniformity, mean anterior tendon displacement, and Achilles tendon shear wave velocity at the proximal measurement location. Sex, sport specialisation, history of lower limb injury, and leg dominance did not affect the measured outcomes. The reliability of SWE measurement was found to range from moderate in soleus muscle to excellent in tendon, which should be considered when interpreting and generalising results.

### Acute exercise effects on tendon displacement

Lower AT non-uniformity and anterior tendon mean displacement were found in athletes who had exercised within two days prior to measurements compared to those who had not exercised. To our knowledge, such a difference in internal AT displacement after exercise has not been previously reported. Correlation analysis showed that the lower displacement of the anterior tendon was not associated with shear modulus of the TS muscles. However, it is important to note that the tendon displacement was measured during active force production and muscle shear modulus under passive tension suggesting that passive muscle shear modulus does not dictate the tendon function during active force production.

Some studies have related the distal anterior tendon displacement to the gastrocnemii subtendons (Khair et al. 2022), while other studies suggest anterior displacement to be related with soleus subtendon (Lehr et al. 2021). Thus, the present observation of lower displacement of anterior tendon may indicate that exercise affects the internal tendon function either via the gastrocnemii muscles or the soleus. This may differ between individuals given the anatomical variability (Pękala et al. 2017). Previous studies have shown that the Achilles tendon exhibits acute biomechanical changes after exercise, such as a decrease in tendon force and stress (Kay and Blazevich 2009; Obst et al. 2013), and the reduced non-uniformity could indicate differential fatigue in TS muscles, for example. Previously, Rosengarten et al. (2015) reported changes in the Achilles tendon echo pattern assessed by ultrasound tissue characterisation two days after maximal exercise in healthy athletes. To date, AT displacement has been only reported in a context of ageing (Slane and Thelen 2015; Franz and Thelen 2016) and injury (Beyer et al. 2018; Khair et al. 2021). Future studies should investigate the specific modes of exercise that lead to the observed changes in internal tendon displacement and whether these changes are related to, for example, muscle fatigue and subsequent reduction in force production capacity.

In the present study, exercise or the time between exercise and the measurement was not controlled, and the actual cause of the observed group difference remains unknown. More information about acute changes in tendon properties after exercise might help develop ways to prevent overuse injuries. A decreased AT non-uniformity pattern has previously been shown after tendon injury (Beyer et al. 2018; Khair et al. 2021) and based on the current findings, AT non-uniformity and anterior tendon displacement were ~ 20% and ~ 13% lower after exercise also in uninjured individuals. Future intervention studies should examine whether there are individual differences in the recovery of the internal tendon sliding to baseline, which could be used to guide training and rehabilitation to reduce the risk of tendon overuse conditions. From a methodological perspective, when assessing individual differences in internal tendon displacement, the possible effect of previous physical exercise prior to laboratory testing should be considered when interpreting the results.

Based on the previous literature, we initially expected SWE findings to differ according to the prior exercise. However, SWV of ATprox was the only SWE imaging parameter that differed between athletes with and without previous exercise. Previous reports have shown that shear wave-based AT stiffness increases after a 30-min moderate intensity run (Payne et al. 2018a), and eccentric heel drop exercise has been observed to increase both AT and gastrocnemii muscle stiffness (Leung et al. 2017). These findings may indicate an individual response to exercise, based on pre-existing mechanical properties and the specific type of sport and loading of the structures involved. A previous study has shown that tendon structure can be altered up to 72 h after exercise when assessed with ultrasound tissue characterisation (Rosengarten et al. 2015). In addition, a short-term decrease in tendon thickness has been found after exercise (Grigg et al. 2009; Fahlström and Alfredson 2010). These changes are thought to be caused by a loss of fluid from the tendon into the peritendinous space due to the mechanical loading and increased hydrostatic pressure. This reversible loss of fluid from the tendon may also explain changes in shear wave velocity after exercise. In the present study, muscle shear modulus was measured during a passive stretch, and it is possible that exercise-induced changes could have been detected without the applied tension, or alternatively, in an active state. In addition, the type and onset of previous exercise was not recorded in detail, which may have led to a wide variation in the type and timing of exercise. Physical exercise that does not substantially load the AT or TS muscles, such as ice skating, swimming, or cycling, may not significantly alter the passive stiffness of these structures compared to several weight-bearing exercises. Furthermore, it is possible that any changes in stiffness had already returned to baseline at the time of measurement, as the exercise-induced increase in AT shear wave velocity has been shown to return to baseline within six hours (Payne et al. 2018a).

### Effect of previous injuries

There were no differences in the measured tendon or muscle parameters between athletes who did or did not report lower limb conditions or symptoms within the previous 6 months.

Previous studies have suggested SWE as a potential tool for detecting and monitoring musculoskeletal injuries (Dirrichs et al. 2016; Laurent et al. 2020; Pan et al. 2021; Crawford et al. 2023). Diseased tendons have been shown to be softer than healthy tendons (Dirrichs et al. 2016; Laurent et al. 2020; Crawford et al. 2023), and this decrease in stiffness has been associated with histological degeneration (Klauser et al. 2013) and clinical symptoms (Dirrichs et al. 2016; Ooi et al. 2016). At the time of measurement in the present study, all athletes were actively training and competing, so previous conditions no longer affected participation in exercise and sport. In this study, the conditions reported by the athletes varied widely, ranging from mild ankle and muscle sprains to lower extremity joint pain. None of the athletes reported AT or TS muscle conditions, but to confirm the integrity of the results obtained, we wanted to identify and outline the possible influence of pre-existing lower limb conditions and symptoms.

### Effect of sport-specific loading

Across the study population, the outcome variables did not differ significantly between sports.

As the sports in this study were not particularly one-side dominant, we did not expect to find bilateral differences. The performance demands and sport-specific requirements of these sports pose different challenges to the musculoskeletal system, which may lead to selective tendon adaptations. Previous biomechanical studies assessing material, morphological, and mechanical properties of tendon have shown differences between limbs in response to asymmetric loading in athletes (Couppé et al. 2008; Bayliss et al. 2016). AT force and stress have been found to be higher in the jump leg of long and high jumpers (Bayliss et al. 2016), and similarly, distal patellar tendon cross-sectional area and tendon stiffness higher in the lead leg of fencers and badminton players (Couppé et al. 2008). Previous studies have reported symmetrical findings in AT SWE assessments (Payne et al. 2018a; Siu et al. 2016), but in contrast, it has also been reported that SWE-based AT stiffness in the dominant leg was higher before a stretching intervention (Chiu et al. 2016). Studies with larger populations are needed to further understand the laterality of findings in different populations, especially as the healthy contralateral limb is often used as a reference in both clinical and research settings to evaluate injury and recovery.

### Sex differences

We found no sex differences in the measured variables. In this study setting, the internal AT displacement measurement was performed with the same absolute, rather low torque of 50 Nm for all participants, which causes the torque to have differential percentage of the expected MVC between the sexes. However, at such a low torque level, the sex differences may not be as clear as at higher torques. Future studies should consider individualising the measurement by associating the data collection with the same relative torque level. Regarding SWE imaging, there is conflicting evidence of differences between sexes in the previous literature. Several previous publications have suggested that passive muscle and tendon stiffness does not differ according to sex (e.g. Chino and Takahashi 2016; Souron et al. 2016; Wakker et al. 2018; Avrillon et al. 2020; Gonzalez et al. 2021); however, a higher triceps surae stiffness has been found in females compared to males in a sample of long-distance runners (Fouré et al. 2022).

### Muscle shear modulus and tendon shear wave velocity

As expected, the shear modulus of each TS muscle increased with passive ankle dorsiflexion, which is consistent with previous studies using similar lower limb joint alignments and measurement locations within the muscles (Maïsetti et al. 2012; Le Sant et al. 2017). Regarding the shear wave velocity of the free AT, previous studies have shown both similar (Payne et al. 2018a, b; Frankewycz et al. 2020; Althoff et al. 2023) and higher (DeWall et al. 2014; Laurent et al. 2020; Pan et al. 2021; Crawford et al. 2023) SWV outcomes compared to the present study. While other groups have used a relaxed ankle position, we imaged the AT in a fixed 25° ankle plantarflexion. The fixed position was chosen to standardise the ankle angle between participants while using a plantarflexion angle corresponding to resting ankle angles reported for the prone position (Pan et al. 2021; Crawford et al. 2023).

Based on animal experiments, it was expected that the AT shear wave velocity would be higher in distal rather than proximal tendon region (Arruda et al. 2006). In humans, several studies using SWE have reported fairly uniform stiffness across different regions of the free tendon (Frankewycz et al. 2020; Laurent et al 2020; Pan et al. 2021; Crawford et al. 2023), while we found a significant increase in shear wave velocity from distal to proximal tendon. To confirm our findings, we further evaluated the differences between imaging sites for subgroups by sex and sport, with the significance of the mean differences remaining. Similar results were recently reported by Althoff and colleagues (2023) in a small sample of athletes and by Laurent et al. (2020) in the unaffected leg of patients recovering from Achilles tendon rupture. Previously, DeWall et al. (2014) reported divergent trend in AT shear wave velocity stiffness when imaged either medially or laterally. The findings of the present study and previous literature about the regional differences in AT shear wave velocity measured using SWE highlight the need for consistency in the selection and reporting of measurement sites. Further research efforts to understand the discrepancy between animal and human SWE studies are needed.

### Measurement reliability

This is the first study to assess the measurement reliability of internal AT displacement. Although the calculated ICC values ranged from moderate to excellent, the other reliability measures showed greater variation between two trials. These results suggest that the assessment may be susceptible to between-trial and between-rater error. For instance, some of the observed MDCs (0.9–4.1 mm) exceeded the reported group differences showing lower AT non-uniformity (1.8 mm) and mean displacement (1.6 mm) in athletes who had exercised within two days prior to measurements. Therefore, the observed group differences need to be confirmed with further studies. In repeated measures, careful probe placement in both longitudinal and sagittal orientation is important to obtain repeatable outcomes. In this study, the laboratory setting for measuring AT displacement was challenging as the ultrasound probe was secured on the tendon for a participant seated on a dynamometer with limited space around the foot. Furthermore, differential torque production during plantarflexion contractions may affect the magnitude of displacement (Franz et al. 2015).

The current literature shows various types of reporting of the measurement reliability using SWE (e.g. Cortez et al. 2016; Laurent et al. 2020), whereas in some studies, these results are lacking (Pan et al. 2021; Crawford et al. 2023; Althoff et al. 2023). Recently, a systematic review by Schneebeli et al. (2021) brought up critical conclusions related to tendon SWE reliability: results range from poor to excellent, and in several cases, the ways of reporting have been inadequate. For instance, the ICC value alone is not sufficient to describe the reproducibility of assessment (Koo and Li 2016), which is also observed in the present study. Through transparent reporting, we aim to point out aspects of caution when using the SWE method and interpreting the results. In particular, when SOL was imaged during the passive stretching manoeuvre, the intrarater reliability was inferior with the CV of peak values ranging from 26 to 40% by two raters. SWE has been recommended as a reliable tool for measuring large, superficial muscles, whereas muscles with complex structure or deep location may be problematic (Creze et al. 2018). The soleus muscle divides into four compartments with individual variation in the location of its distal insertion (Finni et al. 2003) and with ankle movement causing fascicle rotation. The MDC for SOL was ~ 15 kPa, indicating that ~ 65% change would be required for intervention studies to observe a significant change. Similarly, for MG and LG, a minimum change of ~ 22% would be required based on the calculated MDC in this sample. Changes in AT SWE would need to be minimum of 15% the changes to be meaningful. These numbers may help in power calculations of future studies. The present results regarding SWE measurement reliability emphasise the importance to carry out operator reliability assessments in each study, rather than relying on previous studies reporting acceptable reliability for a given device and study protocol.

### Limitations

This study has some limitations. First, surface electromyography was not used to ensure that participants were relaxed during the SWE measurements. Participants were instructed to remain relaxed, and relaxation was observed from the real-time torque signal during the SWE recordings of the TS muscles. During data processing, both SWE and torque data were checked for possible muscle contractions to outline the possible influence on the results. Second, TS muscle SWE measurements were performed consecutively for each muscle belly. Thus, each muscle was successively subjected to a slow passive stretch to ankle dorsiflexion for a total period of approximately 35 s with approximately 1 min of rest periods between muscles. A recent study by Umehara et al. (2021) showed an acute decrease in shear modulus in response to a continuous 30 s muscle stretch. Although the TS muscle stretch was not maintained after reaching the maximum of 20° dorsiflexion, the potential effect of the prior stretch cannot be fully outlined. We considered that randomising the order of MG, LG, and SOL imaging would reduce the influence of the possible stretch effect on the results. Next, it should be noted that the conditions for calculating muscle shear modulus from SWV (see method) may not be fully met, as muscle tissue is not homogeneous and muscle mass density may differ slightly from 1000 kg m^3^. However, we chose to report the shear modulus to allow comparison with previous studies (e.g. Le Sant et al. 2017). Furthermore, as our aim was to analyse the changes in muscle mechanics across the ROM using SWE, it was important to report a modulus rather than SWV, which would have a different relationship with angle due to the square in the formula. Finally, regarding tendon displacement measurements, it must be recognised that ultrasound is two dimensional, and the complex three-dimensional structure of the AT may contribute to the variability seen in the reliability assessment.

## Conclusions and perspective

This study provides new insights into the acute effects of exercise on the internal Achilles tendon displacement pattern, which may be an indicator of the normal recovery process of the tendon. Previous exercise should be considered when assessing internal tendon sliding, and future research should continue to identify the types of exercise, and furthermore, the recovery of the tendon properties to baseline. If individual differences in the delay in recovery of this tendon function were identified, this could serve as an early marker of overuse conditions. The ultrasound imaging techniques used can improve the understanding of the biomechanics of muscles and tendons in different populations with different mechanical characteristics related to functional performance.

### Supplementary Information

Below is the link to the electronic supplementary material.

Supplementary file1 (PDF 118 KB)

## Data Availability

The datasets used in this study are available from the corresponding author on reasonable request.

## Abbreviations

AT: Achilles tendon
ATdist: Achilles tendon, distal SWE measurement location
ATmid: Achilles tendon, middle SWE measurement location
ATprox: Achilles tendon, proximal SWE measurement location
LG: Lateral gastrocnemius
MG: Medial gastrocnemius
SOL: Soleus
SWE: Shear wave elastography
SWV: Shear wave velocity
TS: Triceps surae muscle
