# Correction to: Achilles tendon and triceps surae muscle properties in athletes

**DOI:** 10.1007/s00421-023-05401-2

**Published:** 2023-12-26

**Authors:** Maria Sukanen, Ra’ad M. Khair, Johanna K. Ihalainen, Iida Laatikainen-Raussi, Pauline Eon, Antoine Nordez, Taija Finni

**Affiliations:** 1 Faculty of Sport and Health Sciences, University of Jyväskylä, Jyväskylä, Finland; 2 Nantes Université, Movement-Interactions-Performance, MIP, UR 4334, 44000 Nantes, France; 3 Institut Universitaire de France, Paris, France

**Correction to: European Journal of Applied Physiology** 10.1007/s00421-023-05348-4

The original version of this article unfortunately contained a mistake. The corrected detail is provided below:

In the abstract, results and conclusion, which previously read:

**Results** A total of 131 athletes participated in this study. Athletes who had not exercised within two days had greater AT nonuniformity and mean anterior tendon displacement, and lower SWV at the proximal AT measurement site (mean difference [95% CI]: 1.8 mm [0.6–2.9], *p* = 0.003; 1.6 mm [0.2–2.9], *p* = 0.021; − 0.9 m × s^−1^ [− 1.6 to − 0.2], *p* = 0.014, respectively). Male basketball players had a lower mean AT displacement compared to gymnasts (− 3.7 mm [− 6.9 to − 0.5], *p* = 0.042), with the difference localised in the anterior half of the tendon (− 5.1 mm [− 9.0 to − 1.1], *p* = 0.022). Male gymnasts had a smaller absolute difference in medial gastrocnemius-minus-soleus shear modulus than basketball players (59.6 kPa [29.0–90.2], *p* < 0.001) and track and field athletes (52.7 kPa [19.2–86.3], *p* = 0.004). Intraclass correlation coefficients of measurements ranged from 0.720 to 0.937 for internal AT displacement, from 0.696 to 0.936 for AT SWE, and from 0.570 to 0.890 for TS muscles.

**Conclusion** This study provides a reliability assessment of muscle and tendon SWV. The relative differences in passive TS muscle shear modulus suggest sport-specific adaptation. Importantly, in healthy individuals, lower AT displacement after exercise may reflect the time required for tendon recovery.

Should read:

**Results** A total of 131 athletes participated in this study. Athletes who had not exercised within two days had greater AT nonuniformity and mean anterior tendon displacement, and lower SWV at the proximal AT measurement site (mean difference [95% CI]: 1.8 mm [0.6–2.9], *p* = 0.003; 1.6 mm [0.2–2.9], *p* = 0.021; − 0.9 m × s^−1^ [− 1.6 to − 0.2], *p* = 0.014, respectively). No statistical differences were found based on sex, sport specialisation, leg dominance or history of lower limb injury. Intraclass correlation coefficients of measurements ranged from 0.720 to 0.937 for internal AT displacement, from 0.696 to 0.936 for AT SWE, and from 0.570 to 0.890 for TS muscles.

**Conclusion** This study provides a reliability assessment of muscle and tendon SWV. The results suggest similar internal AT displacement and SWE imaging outcomes between males and females. Importantly, in healthy individuals, lower AT displacement after exercise may reflect the time required for tendon recovery.

