# Supplementary material for: Achilles tendon and triceps surae muscle properties in athletes
Source: Eur J Appl Physiol. 2023 Nov 11;124(2):633–47. doi: 10.1007/s00421-023-05348-4 (PMC10858159; doi:10.1007/s00421-023-05348-4)
Supplement: Supplementary file 1 — Supplementary file1 (PDF 118 KB) [file 421_2023_5348_MOESM1_ESM.pdf]

Achilles tendon and triceps surae properties in athletes
European Journal of Applied Physiology
Maria Sukanen, Ra'ad Khair, Johanna K. Ihalainen, Iida Laatikainen-Raussi, Pauline Eon, Antoine Nordez, Taija Finni
Corresponding author: Maria Sukanen, Faculty of Sport and Health Sciences, University of Jyväskylä, Jyväskylä, Finland
maria.e.sukanen@jyu.fi

Supplementary information 1. Descriptive values of triceps surae muscle shear modulus (kPa) and Achilles tendon shear wave velocity (m×s⁻¹).

	MG peak stiffness		LG peak stiffness		SOL peak stiffness		ATdist mean stiffness		ATmid mean stiffness		ATprox mean stiffness	
Subgroup	kPa (SD)	$m \times s^{-1}$ (SD)	kPa (SD)	$m \times s^{-1}$ (SD)	kPa (SD)	$m \times s^{-1}$ (SD)	kPa (SD)	$m \times s^{-1}$ (SD)	kPa (SD)	$m \times s^{-1}$ (SD)	kPa (SD)	$m \times s^{-1}$ (SD)
All athletes	90.7 (23.3)	9.3 (1.2)	53.4 (16.4)	7.1 (1.1)	22.9 (13.4)	4.4 (1.0)	60.2 (32.2)	7.5 (1.8)	93.3 (46.2)	9.3 (2.3)	121.9 (44.8)	10.7 (2.0)
Males	87.3 (22.4)	9.0 (1.2)	53.4 (16.4)	7.1 (1.1)	21.5 (13.7)	4.4 (1.3)	64.0 (36.5)	7.6 (2.0)	101.8 (52.1)	9.6 (2.6)	120.7 (50.8)	10.6 (2.3)
Females	92.7 (23.8)	9.4 (1.2)	53.5 (16.5)	7.1 (1.1)	23.5 (13.3)	4.5 (0.9)	57.7 (29.1)	7.3 (1.6)	88.4 (41.9)	9.1 (2.1)	122.5 (41.3)	10.8 (1.8)
Sport												
Football	93.8 (22.5)	9.5 (1.2)	55.3 (14.3)	7.3 (1.0)	27.5 (17.3)	4.7 (0.7)	65.4 (39.3)	7.8 (2.0)	80.0 (38.51)	8.6 (1.9)	117.1 (37.6)	10.6 (1.7)
Ice hockey	90.3 (28.8)	9.2 (1.5)	54.5 (17.9)	7.2 (1.1)	21.0 (10.9)	4.4 (1.1)	49.4 (19.1)	6.8 (1.3)	88.6 (41.6)	9.1 (2.1)	121.6 (40.2)	10.8 (1.8)
Basketball	90.3 (21.9)	9.1 (1.3)	56.7 (16.1)	7.3 (1.1)	18.4 (9.1)	4.1 (1.0)	64.0 (37.4)	7.6 (2.0)	95.8 (51.7)	9.3 (2.6)	119.5 (56.0)	10.5 (2.5)
Track and field	89.2 (19.7)	9.3 (1.0)	47.1 (15.7)	6.7 (1.1)	20.9 (10.5)	4.3 (1.1)	65.3 (33.2)	7.8 (1.9)	110.0 (51.8)	10.0 (2.4)	138.6 (43.2)	11.4 (1.9)
Gymnastics	89.0 (23.7)	9.2 (1.27)	52.3 (17.8)	7.1 (1.2)	25.0 (15.2)	4.7 (1.4)	58.0 (27.8)	7.3 (1.7)	98.4 (46.4)	9.5 (2.3)	117.7 (45.7)	10.5 (2.2)

MG, medial gastrocnemius; LG, lateral gastrocnemius; SOL, soleus; ATdist, ATmid, and ATprox, distal, middle, and proximal Achilles tendon measurement site; kPa, kilopascal; SD, standard deviation
